# Discussion of Dr. McLaren’s Paper at the Meeting of the I. H. A.

**Published:** 1892-03

**Authors:** 


### Discussion of Dr. McLaren's Paper at the Meeting of the I. H. A., Wednesday Evening, June 24th, 1891.

Dr. Kennedy—I should like to ask the Doctor if he was influenced very much by the character of the convulsions or the time of their occurrence in the twenty-four hours.

Dr. McLaren—The peculiarity of both cases of the boys was that they were taken with a convulsion when asleep and at urination. A fit occurring during sleep without urination is Calcarea. Bufo. and Silicea have similar symptoms.

Dr. Kimball—I have a case getting along beautifully on Bufo. I think Lachesis also has the convulsion during sleep.

Dr. Kent—Did the attacks pass through lighter stages into epileptiform vertigo, or did they cease suddenly?

Dr. McLaren—One passed through a series of light attacks and finally into a chorea. Another had long attacks of vertigo in the place of the convulsions; during these she was very cross and spiteful.

Dr. Kent—It has been my experience that when epilepsy is cured it passes away in lighter and lighter attacks, finally to a vertigo which remains a long time. I mentioned one a year ago cured by Silicea. This patient remains entirely well, never having a fit, but suffering slight attacks of vertigo every few days. During an attack, if driving, he will turn the buggy around and then catch himself going the other way, the attack lasting a minute, or possibly two minutes. We have recently had a case in the clinic in Philadelphia of great interest, but it is too soon to claim any permanent benefit.

It is a case that had been under treatment for many years. He received a dose of Sulphur and since that has gone on for two months without any fit. He has, however, the vertigo, probably ten attacks in the course of a few weeks, but they are light. He says he simply feels strange at times. A large abscess formed over the right hip, and another one appears to be forming over the left hip. This is regarded as a manifestation of the action of Sulphur. I think it is going to be a very interesting case.

Dr. H. C. Allen—I have seen a number of cases of epilepsy and have cured a few, but I have never succeeded in curing a case that had previously been subjected to the action of the bromides. I should like to know whether any member of the Association has cured or seen cured a case so subjected.

Dr. McLaren—I saw one case, which was hardly epilepsy, but spasms or convulsions occurring in an infant. The mother was dosed with bromides before the child was born. The child showed nervous symptoms as soon as born. I discovered that the attacks came on out-of-doors, and that the child clung tightly to the nurse; it was cured very completely with Borax. First the 200th was given, and when the fits returned the CM.

Dr. Kimball—I have a case that was subjected to the action of Bromide of Potassium three times a day for a year. I have prescribed for her. It is too soon to say she is cured, but the improvement in general is marked, and convulsions are getting lighter and less frequent. She used to have the convulsions every month. After beginning the Bromide she had no more convulsions, but a pustular eruption. When I stopped the Bromide she had convulsions every week, but gained greatly in general health, and the pustules disappeared.

Dr. McLaren—Do the bromides affect the system so deeply that it cannot be rid of them?

Dr. H. C. Allen—I am not prepared to explain what it does or how it makes the cases so difficult of cure, but I have observed the fact. I have in a number of such cases been able to delay or postpone the paroxysms but never to make a complete cure.

Dr. Kimball—Perhaps some of the members will recollect a case of epilepsy that I reported last year. It was in a woman of forty-four years of age, and you may recall the remedy prescribed, which was Lachesis. She had one convulsion in December, one in July, and one since she came to me in September. The medicine was given in April.

